# Supplementary material for: Modulation of MRSA virulence gene expression by the wall teichoic acid enzyme TarO
Source: Nat Commun. 2023 Mar 22;14:1594. doi: 10.1038/s41467-023-37310-5 (PMC10032271; doi:10.1038/s41467-023-37310-5)
Supplement: Supplementary file 1 — Supplementary Information [file 41467_2023_37310_MOESM1_ESM.pdf]

Supplementary Information for

Modulation of MRSA virulence gene expression by the wall teichoic acid enzyme TarO

Yunfu Lu[1,2,3#], Feifei Chen[1,2,4#], Qingmin Zhao[1,2,3], Qiao Cao[1,2,4], Rongrong Chen[1,2] Huiwen Pan[1], Yanhui Wang[1,2,3], Haixin Huang[2], Ruimin Huang[2,3], Qian Liu[5], Min Li[5], Taeok Bae[6], Haihua Liang[4,7*], Lefu Lan[1,2,3,4*]

[#]Y.L. and F.C. contributed equally to this work.

*To whom correspondence should be addressed, Email: llan@ucas.ac.cn (L.L.) or lianghh@sustech.edu.cn (H. L)

This file includes:

Figures S1 to S10

Tables S1 to S3

SI References

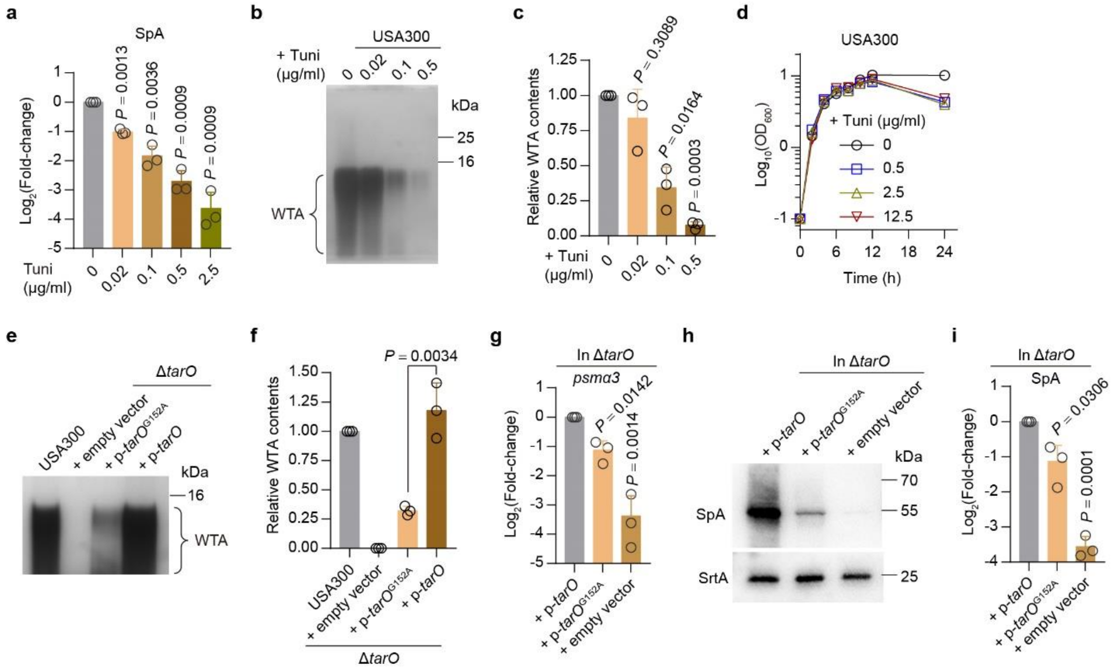

Figure S1. WTA contents positively associate with the expression levels of *psmα* and SpA in USA300 LAC. (**a**) Quantitative analysis of SpA in *S. aureus* strains grown in TSB medium supplemented with or without tunicamycin for 3 h. Data represent mean ± SD from *n* = 3 independent experiments. (**b** and **c**) Representative images of PAGE analysis (**b**) and quantitative analysis (**c**) of WTAs from USA300 LAC strain treated with tunicamycin for 3 h. Data represent mean ± SD from *n* = 3 independent experiments. (**d**) Effect of tunicamycin on the growth of USA300 LAC in TSB medium. Data represent mean ± SD from *n* = 3 biological replicates. (**e** and **f**) Representative images of WTA PAGE analysis (**e**) and quantitative analysis (**f**) of *S. aureus* strains grown in TSB medium for 3 h. Results are reported as fold changes compared with WT USA300. Data represent mean ± SD from *n* = 3 independent experiments. (**g**) qRT-PCR analysis of *psmα3* transcripts in *S. aureus* strains grown in TSB medium for 3 h, presented as relative expression levels (log2 fold changes) compared with the control sample (i.e., Δ*tarO* carrying p-*tarO)*. Data represent mean ± SD from three

independent experiments. (**h** and **i**) Representative images (**h**) and quantitative analysis (**i**) of Western blotting for SpA in *S. aureus* strains grown in TSB medium for 3 h. SrtA is a loading control, and the results are reported as relative expression levels (log2 fold changes) compared with control sample (i.e., Δ*tarO* carrying p-*tarO*). Data represent mean ± SD from *n* = 3 independent experiments. In (**e**) to **i**), USA300 LAC harbors an empty pYJ335-1 vector as control, Δ*tarO* carries either an empty pYJ335-1 vector or a p-*tarO* plasmid without (+ p-*tarO*) or with G152A missense mutation in *tarO* (+ p-$tarO^{G152A}$). Statistical analysis was performed using two-tailed one-sample *t*-test (in **a** and **c**, with the untreated WT USA300 set to a fold change of 1; in **g** and **I**, with Δ*tarO* carrying p-*tarO* set to a fold change of 1) and Student's two-tailed unpaired *t*-test (in **f**).

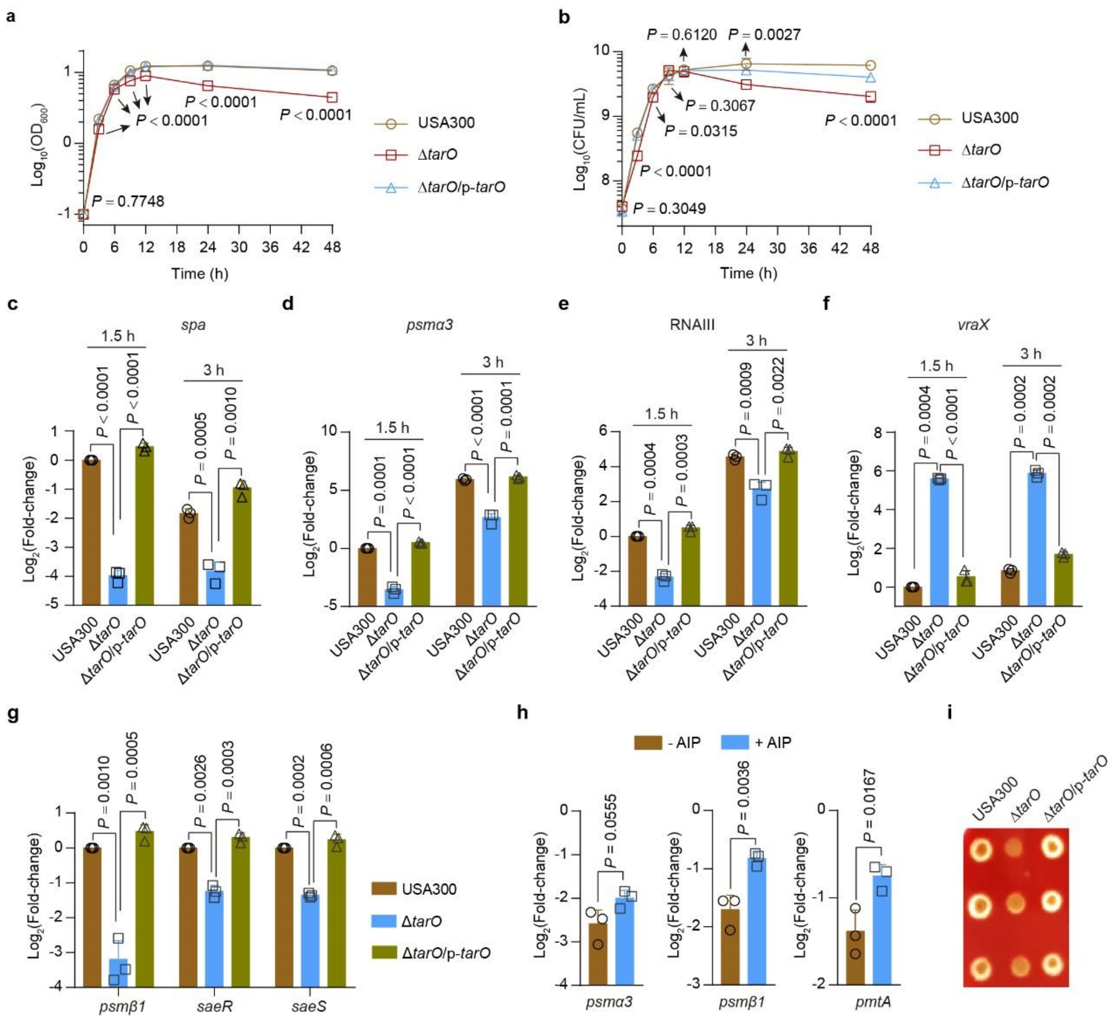

Figure S2. Effect of *tarO* deletion on the growth, the expression of virulence gene, and the hemolytic activity of USA300 LAC. (**a**) Growth curve of *S. aureus* strains in TSB medium. Data represent mean ± SD from *n* = 4 biological replicates. (**b**) Growth of *S. aureus* strains measured by CFU/mL quantification. Data represent mean ± SD from *n* = 4 biological replicates. CFU, colony forming unit. (**c** to **f**) qRT-PCR analysis of *spa* (**c**), *psmα3* (**d**), RNAIII (**e**), and *vraX* (**f**) in *S. aureus* strains grown in TSB medium for 1.5 h and 3 h, presented as relative expression (log2 fold changes) compared with the control sample (WT USA300 at 1.5-h time point). Data represent mean ± SD from three independent experiments. (**g**) qRT-PCR analysis of *psmβ1*, *saeR*, and *saeS* in *S. aureus* strains grown in TSB medium for 3 h, presented as relative expressions (log2

fold changes) compared with the control sample (WT USA300). Data represent mean ± SD from three independent experiments. (**h**) Effect of the exogenous addition of AIP (1.5 µM) on the expression levels of *psmα3*, *psmβ1,* and *pmtA* in Δ*tarO* mutant cultured in TSB medium for 3 h. Results presented as relative expression levels (log2 fold changes) compared with the control sample (WT USA300). Data from n = 3 biological replicates are reported as the mean ± SD. (**i**) Hemolytic activity of *S. aureus* strains on the sheep blood agar plate. In all panels, USA300 and Δ*tarO* strains harbor an empty pYJ335-1 vector as control, Δ*tarO*/p-*tarO* denotes the complemented strain of Δ*tarO* (Table S2). Statistical analysis was performed using Student's two-tailed unpaired *t*-test (in **a** and **b**, Δ*tarO* versus WT USA300 LAC; in **h**) and two-tailed one-sample *t*-test (in **c** to **f,** when compared with the WT USA300 at 1.5-h time point, which was set to a fold change of 1; in **g**, when compared with the WT USA300, which was set to a fold change of 1) or otherwise with Student's two-tailed unpaired *t*-test (in **c** to **g**).

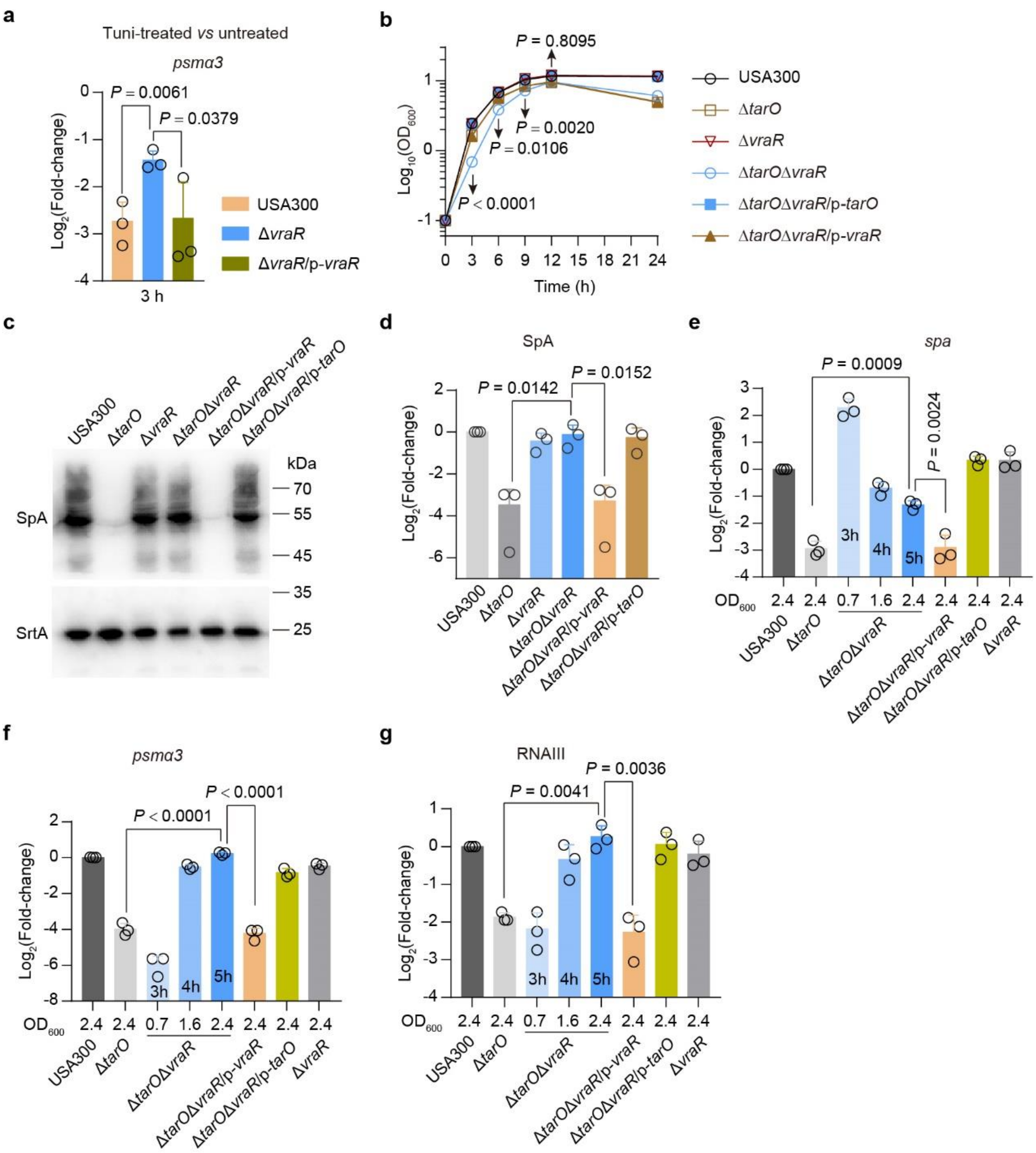

Figure S3. VraR mediates TarO inhibition-induced changes in gene expression. (**a**) qRT-PCR analysis of the effect of tunicamycin (0.5 µg/ml) treatment on the expression of *psmα3* in *S. aureus* strains grown in TSB medium for 3 h. Results are showed as relative expressions (log2 fold changes) compared with the untreated control. Data represent mean ± SD from three independent experiments. Statistical analysis was performed using Student's two-tailed unpaired *t*-test. (**b**) Growth curve of *S. aureus* strains cultured in TSB medium. The cultures were shaken (250 rpm) at

37°C and the optical density at 600 nm was examined. Data represent mean ± SD from n = *3* biological replicates. Statistical analysis was performed using Student's two-tailed unpaired *t*-test (Δ*tarO*Δ*vraR* versus Δ*tarO*). (**c** and **d**) Representative images (**c**) and quantitative analysis (**d**) of western blotting for SpA in *S. aureus* strains grown in TSB medium for 3 h. SrtA is loading controls, and the results are reported as relative expression levels (log2 fold changes) compared with the WT USA300 control. Data represent mean ± SD from *n* = 3 independent experiments. Statistical analysis was performed using Student's two-tailed unpaired *t*-test. (**e** to **g**) qRT-PCR analysis of the expression of *spa* (**e**), *psmα3* (**f**) and RNAIII (**g**) in *S. aureus* strains grown in TSB medium. Results are showed as relative expressions (log2 fold changes) compared with the WT USA300 control. Data represent mean ± SD from three independent experiments. Statistical analysis was performed using Student's two-tailed unpaired *t*-test. In all panels, USA300, Δ*tarO*, Δ*vraR*, or Δ*tarO*Δ*vraR* mutant harbors an empty pYJ335-1 vector as control.

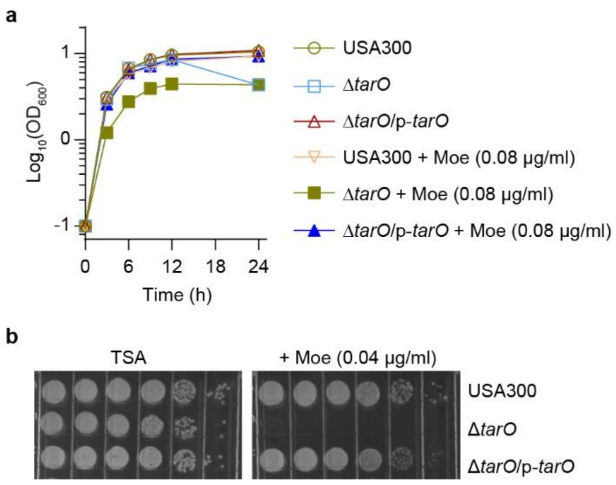

Figure S4. Effect of *tarO* deletion on the susceptibility of USA300 LAC to moenomycin. (**a**) Growth curve of *S. aureus* strains in a 50-ml tube containing TSB medium supplemented with or without moenomycin. The cultures were shaken (250 rpm) at 37°C and the optical density at 600 nm was examined. Data represent mean ± SD from n = *3* biological replicates. USA300 and Δ*tarO* strains harbor an empty pYJ335-1 vector as control, Δ*tarO*/p-*tarO* denotes the complemented strain of Δ*tarO* (Table S2). (**b**) The *tarO* null mutant strain shows decreased resistance to moenomycin compared with either WT USA300 LAC strain (harboring pYJ335-1) or the complemented strain of Δ*tarO* (i.e., Δ*tarO*/p-*tarO*). For this assay, the diluted overnight cultures were grown in TSB medium at 37°C for 3 h, then serial 10-fold dilutions of bacterial suspension were prepared and spotted (10 µl per spot) onto TSA agar plates supplemented with or without moenomycin, and the plates were cultured at 30 °C for 24 h. Images are representative of two independent experiments.

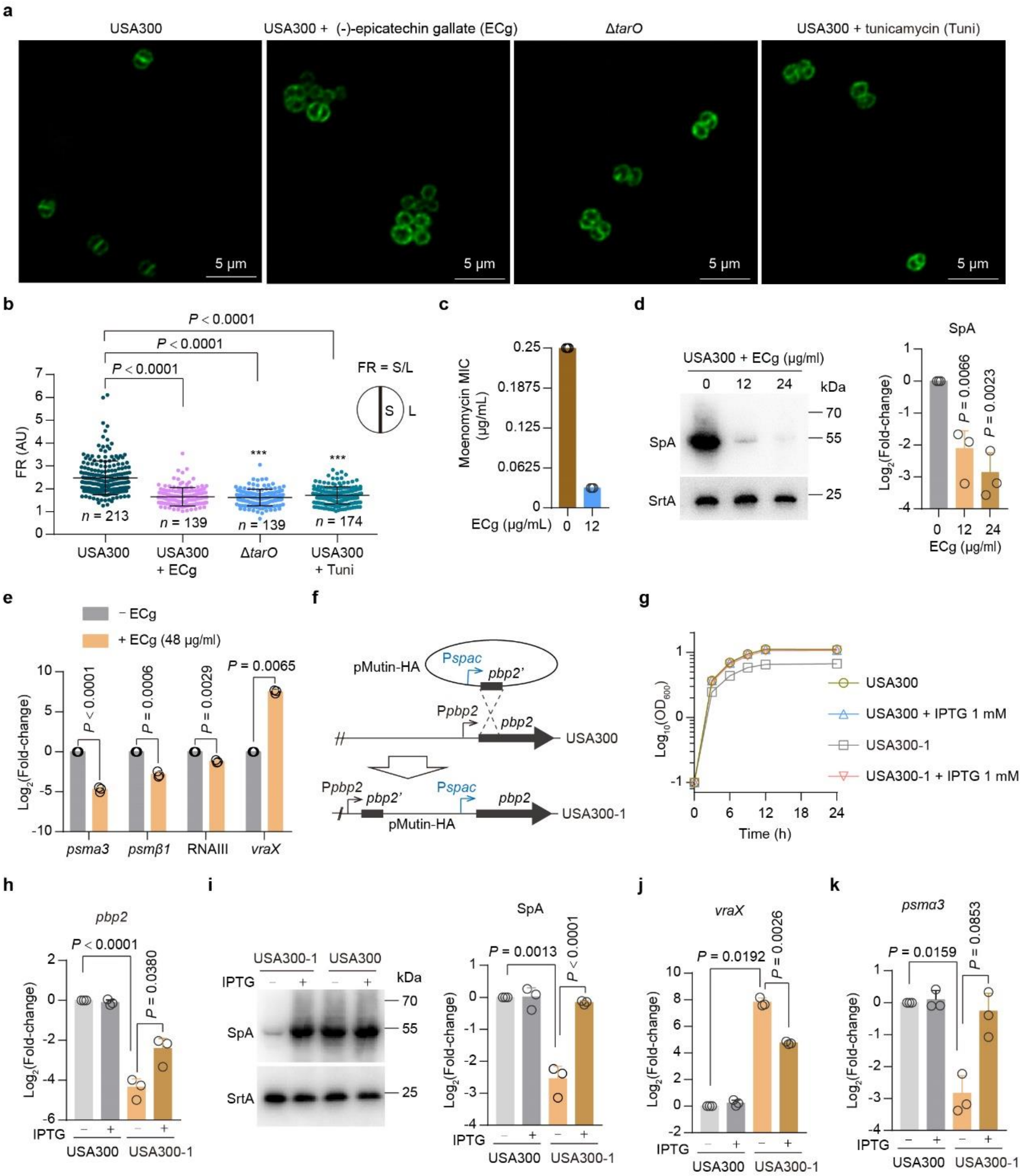

Figure S5. Role of PBP2 in the expression of virulence genes in USA300. (**a**) Effect of ECg treatment, *tarO* deletion, or tunicamycin treatment on the septal localization of GFP-PBP2. The scale bar is 5 µm. (**b**) Quantitative analysis of GFP-PBP2 fluorescence at septum (S) *versus* lateral (L) membrane. Data represent mean ± SD. (**c**) Minimum inhibitory concentrations (MIC) of moenomycin against WT USA 300 LAC in the absence or presence of ECg. (**d**) Representative images (*left panel*) and quantitative analysis (*right panel*) of SpA in WT USA300 strain grown in TSB medium

supplemented with ECg for 3 h. SrtA is loading controls. Data represent mean ± SD from n = 3 independent experiments. (**e**) Effect of ECg treatment on expression of *psmα3*, *psmβ1*, RNAIII, and *vraX*. Data represent mean ± SD from three independent experiments. (**f**) Schematic of construction of *pbp2*-depleted mutant USA300-1. (**g**) Growth curves of USA300 and USA300-1 cultured in TSB medium supplemented with or without IPTG. (**h**) qRT-PCR analysis of *pbp2* transcripts in *S. aureus* strains grown in TSB medium supplemented with (+) or without (-) IPTG (1 mM) for 3 h. Data represent mean ± SD from three independent experiments. (**i**) Representative images (*left panel*) and quantitative analysis (*right panel*) of Western blotting for SpA in *S. aureus* grown in TSB medium supplemented with (+) or without (-) IPTG (1 mM) for 3 h. SrtA is loading controls. Data represent mean ± SD from n = *3* independent experiments. (**j** and **k**) Relative expression levels of *vraX* (**j**) and *psmα3* (**k**) in *S. aureus* strains grown in TSB medium supplemented with (+) or without (-) IPTG (1 mM) for 3 h. Data represent mean ± SD from three independent experiments. Statistical analysis was performed using two-tailed one-sample t-test (in **d** and **e**, when compared with the untreated WT USA300, which was set to a fold change of 1; in **h** to **k**, when compared with the WT USA300 in the absence of IPTG, which was set to a fold change of 1) or otherwise with Student's two-tailed unpaired t-test (in **b,** and **h** to **k**).

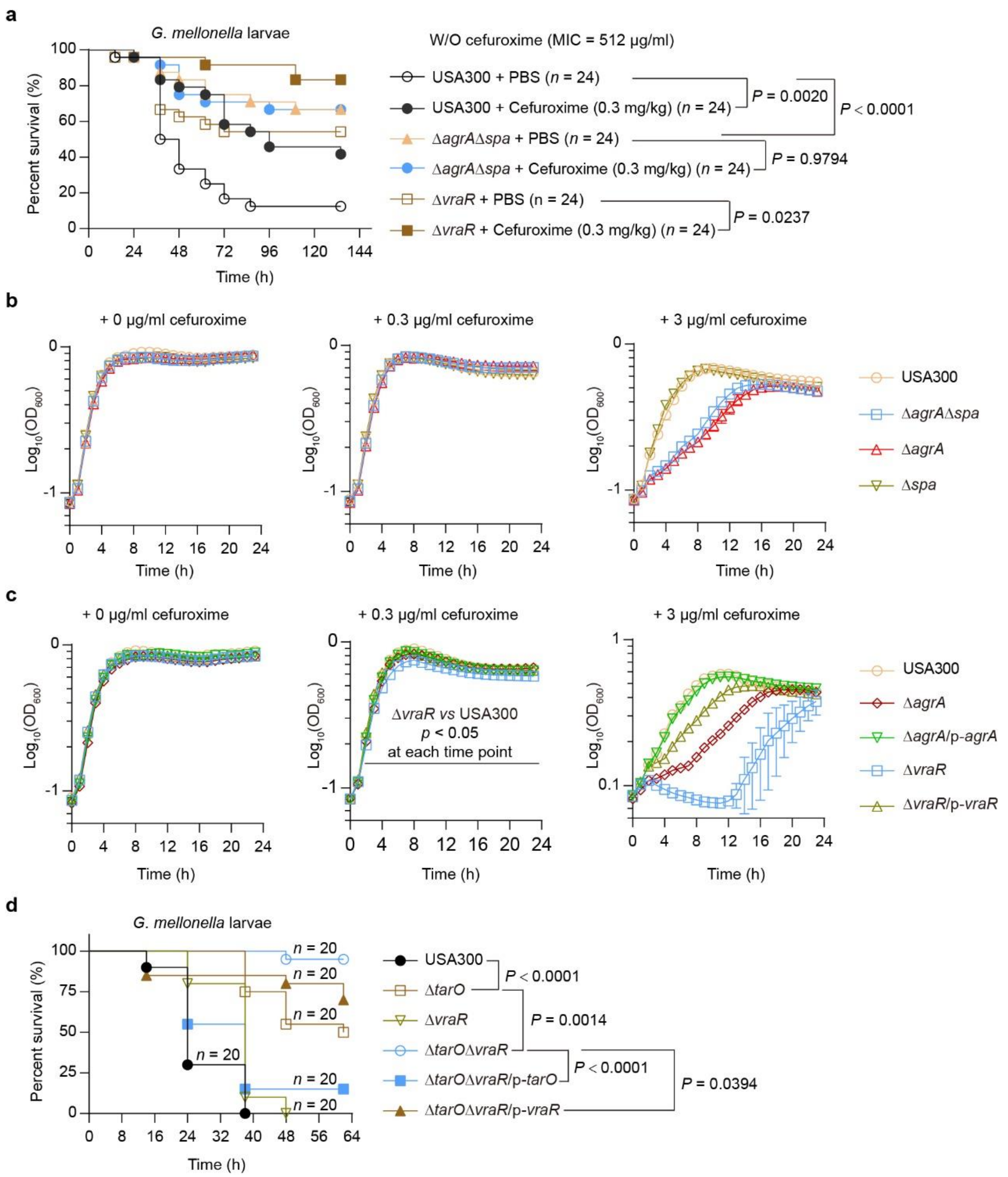

Figure S6. The virulence of *S. aureus* strains against *G. mellonella* larvae and the bacterial growth curves. (**a**) Effect of cefuroxime on the survival rates of *G. mellonella* larvae infected by indicated *S. aureus* strains. *n* indicates the number of *G. mellonella* larvae used. Statistical analysis was performed using log-rank test. (**b** and **c**) The growth curve of *S. aureus* strains cultured in 96-well microtitre plate at 37°C. Overnight cultures were diluted to an $OD_{600} \approx 0.05$ in fresh TSB with or without

cefuroxime (as indicated). Then, 200 µl aliquot of the sample was distributed to a 96-well plate, and 50 µl saxoline was added to each well to prevent evaporation. OD_{600} was monitored at 37℃ using a Synergy 2 Multi-Mode Microplate Reader (Biotek) with 1 h interval. In (**c**), WT USA300, Δ*agrA* or Δ*vraR* harbor an empty pYJ335-1 vector as control. Δ*agrA*/p-*agrA* denotes Δ*agrA* mutant carrying plasmid p-*agrA*; Δ*vraR*/p-*vraR* denotes Δ*vraR* mutant carrying plasmid p-*vraR* . *P < 0.05 by Student's two-tailed unpaired *t*-test (Δ*vraR* versus either WT USA300 or Δ*vraR*/p-*vraR*). (**d**) Survival rates of *G. mellonella* larvae upon infection by indicated *S. aureus* strains. *n* indicates the number of *G. mellonella* larvae used. Statistical analysis was performed using log-rank test. WT USA300, Δ*tarO*, Δ*vraR*, and Δ*tarO*Δ*vraR* harbor an empty pYJ335-1 vector as control. Δ*tarO*Δ*vraR*/p-*tarO* denotes Δ*tarO*Δ*vraR* mutant carrying plasmid p-*tarO*; Δ*tarO*Δ*vraR*/p-*vraR* denotes Δ*tarO*Δ*vraR* mutant carrying plasmid p-*vraR* (Table S2).

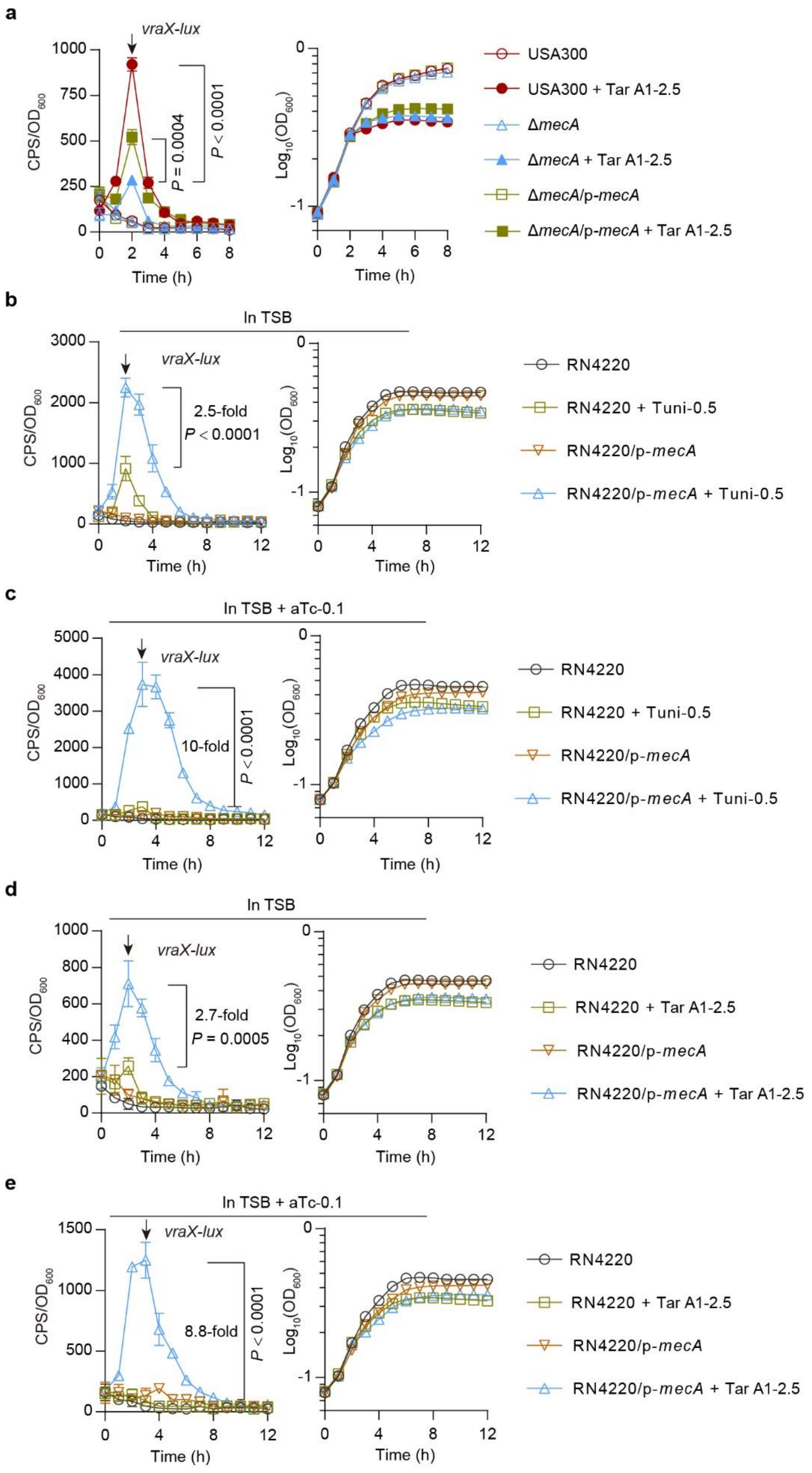

Figure S7. Role of *mecA* in mediating the induction of *vraX-lux* upon TarO inhibition. (**a**) Effect of *mecA* deletion on the expression of *vraX-lux* in USA300 LAC

grown in TSB medium supplemented with either DMSO vehicle control or TarO inhibitor Tarocin A1. Tar A1-2.5, Tarocin A1 at a final concentration of 2.5 μg/ml. Data from *n* = 4 biological replicates are reported as the mean ± SD. Statistical analysis was performed using Student's two-tailed unpaired *t*-test (Δ*mecA* versus either WT USA300 LAC or Δ*mecA*/p-*mecA* when cultured in TSB medium in the presence of Tarocin A1 for 2 h indicated by arrow). (**b** and **c**) Expression of *vraX-lux* in RN4220 derivatives grown in TSB medium (**b**) or in TSB medium supplemented with aTc (**c**) in the presence or absence of tunicamycin. aTc-0.1, aTc at a final concentration of 0.1 μg/ml; Tuni-0.5, tunicamycin at a final concentration of 0.5 μg/ml. Data from *n* = 4 biological replicates are reported as the mean ± SD. Statistical analysis was performed using Student's two-tailed unpaired *t*-test (RN4220/p-*mecA* versus RN4220 when cultured in the presence of tunicamycin for 2 or 3 h, as indicated by the arrows). (**d** and **e**) Expression of *vraX-lux* in RN4220 derivatives grown in TSB medium (**d**) or in TSB medium supplemented with aTc (**e**) in the presence or absence of Tarocin A1. aTc-0.1, aTc at a final concentration of 0.1 μg/ml; Tar A1-2.5, Tarocin A1 at a final concentration of 2.5 μg/ml. Data from *n* = 4 biological replicates are reported as the mean ± SD. Statistical analysis was performed using Student's two-tailed unpaired *t*-test (RN4220/p-*mecA* versus RN4220 when cultured in the presence of Tarocin A1 for 2 or 3 h, as indicated by the arrows). In (**a**) to (**f**), USA300, Δ*mecA* or RN4220 harbors an empty pYJ335 vector as control.

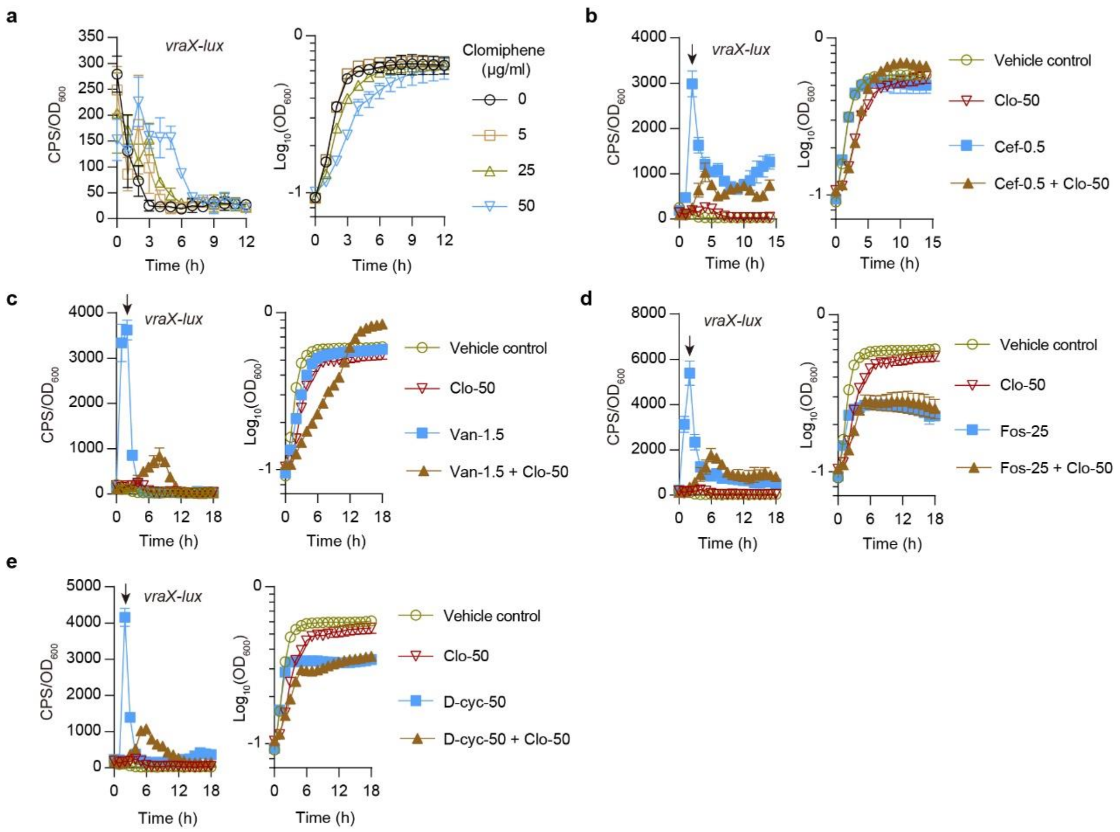

Figure S8. Effect of clomiphene on the expression of *vraX-lux*. (**a**) Effect of clomiphene treatment on the expression of *vraX-lux* in WT USA300 LAC strain. Data from *n* = 3 biological replicates are reported as the mean ± SD. (**b** to **e**) Effect of clomiphene treatment on the *vraX-lux* induction by cefuroxime (**b**), vancomycin (**c**), fosfomycin (**d**), and D-cycloserine (**e**). Cef-0.5, cefuroxime at a final concentration of 0.5 μg/ml; Van-1.5, vancomycin at a final concentration of 1.5 μg/ml; Fos-25, fosfomycin at a final concentration of 25 μg/ml; D-cyc-50, D-cycloserine at a final concentration of 50 μg/ml; clomiphene-50, clomiphene at a final concentration of 50 μg/ml. Data from *n* = 4 biological replicates are reported as the mean ± SD. Arrow indicates the maximal induction of *vraX-lux* under the testing conditions.

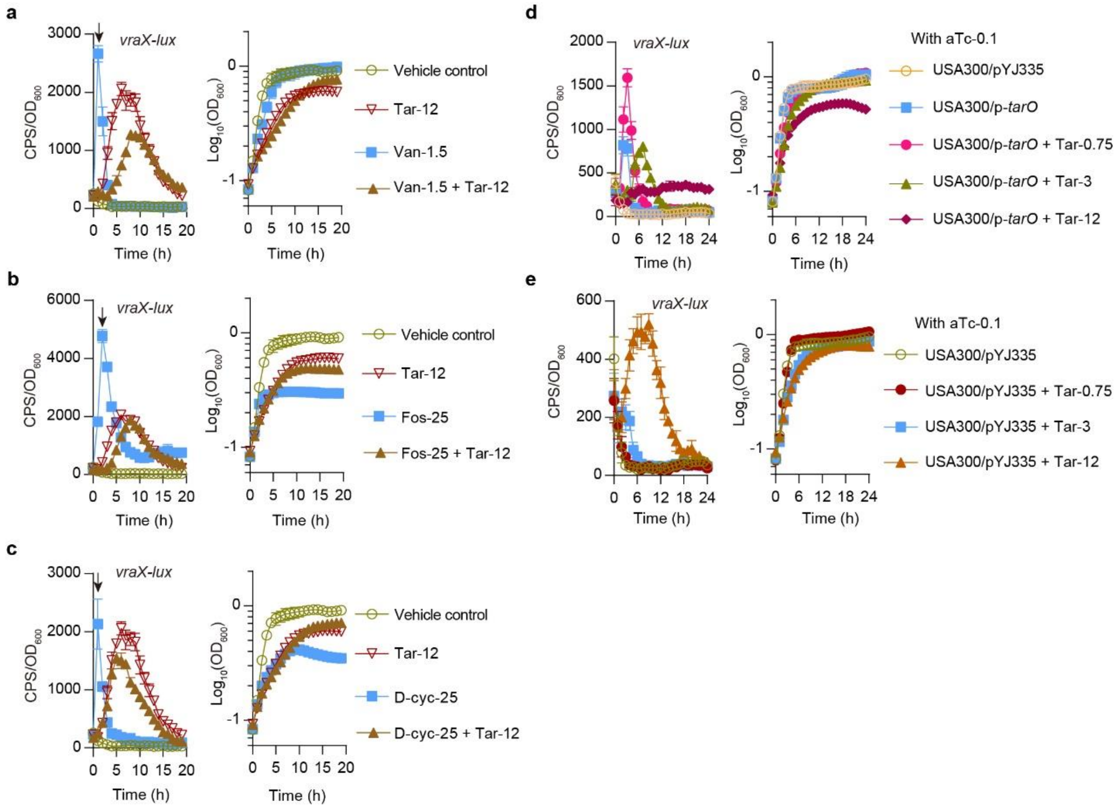

Figure S9. Effect of targocil or *tarO* overexpression on the expression of *vraX-lux*. (**a** to **c**) Effect of targocil treatment on the *vraX-lux* induction in USA300 LAC strain by vancomycin (**a**), fosfomycin (**b**), and D-cycloserine (**c**). Van-1.5, vancomycin at a final concentration of 1.5 µg/ml; Fos-25, fosfomycin at a final concentration of 25 µg/ml; D-cyc-25, D-cycloserine at a final concentration of 25 µg/ml; Tar-12, targocil at a final concentration of 12 µg/ml. Data from n = 4 biological replicates are reported as the mean ± SD. (**d** and **e**) Effect of *tarO* overexpression on the expression of *vraX-lux* in USA300 LAC derivatives grown in TSB medium supplemented with or without targocil in the presence of anhydrotetracycline (aTc-0.1, anhydrotetracycline at a final concentration of 0.1 µg/ml) that induces the tetracycline-inducible *xyl*/*tetO* promoter in the pYJ335 plasmid. The effect of targocil on the expression of *vraX-lux* in USA300/pYJ335 strain is also shown in (**e**). Tar-0.75, Tar-3, and Tar-12 denote targocil

at a final concentration of 0.75, 3, and 12 μg/ml, respectively. Data from $n = 4$ biological replicates are reported as the mean ± SD.

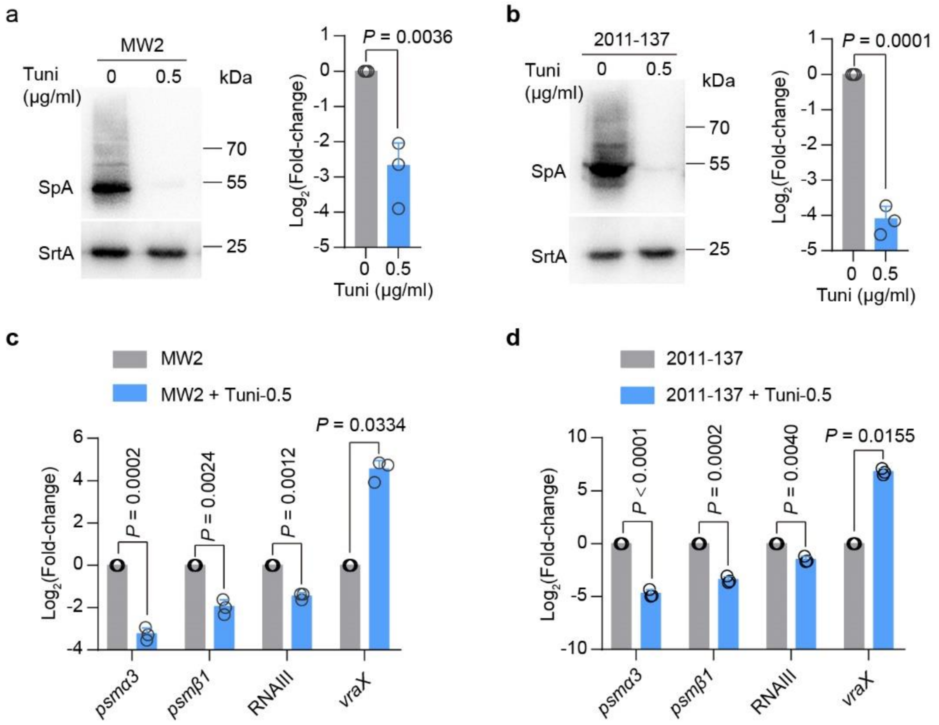

Figure S10. Effect of tunicamycin treatment on virulence gene expression in CA-MRSA isolates USA400 MW2 and 2011-137. (**a** and **b**) Representative images (*left panel*) and quantitative analysis (*right panel*) of Western blotting for SpA in USA400 MW2 (**a**) and 2011-137 (**b**) strains grown in TSB medium supplemented with or without tunicamycin (Tuni). Sortase A (SrtA) is a loading control. Western blot band intensity of SpA was normalized to the intensities obtained with SrtA, and the results are reported as log2 fold changes with the untreated control sample was set to a fold change of 1. Data represent mean ± SD from n = *3* independent experiments. Statistical analysis was performed using two-tailed one-sample *t*-test. (**c** and **d**) qRT-PCR analysis of *psmα3*, *psmβ1*, RNAIII, and *vraX* transcripts in USA400 MW2 (**c**) and 2011-137 (**c**) strains grown in TSB medium supplemented with or without tunicamycin (0.5 μg/ml), presented as relative expression (log2 fold changes) compared with the untreated controls. Data represent mean ± SD from three

independent experiments. Statistical analysis was performed using two-tailed one-sample *t*-test.

Table S1. Effect of tunicamycin on the antibiotic resistance of CA-MRSA strains

Antibiotics	USA300 (-/+)[a]	FC[b]	USA400 (-/+)[a]	FC[b]	2011-137 (-/+)[a]	FC[b]
Cefotaxime	64/4	16	32/4	8	32/4	8
Cefuroxime	512/4	128	32/2	16	8/1	8
Ceftizoxime	>512/16	>32	512/8	64	128/4	32
Moenomycin	0.25/0.0625	4	0.125/0.03125	4	0.25/0.0625	4

[a]Number indicates the minimum inhibitory concentrations (MIC) (µg/ml), " -" and "+" respectively indicates in the absence and presence of tunicamycin (0.5 µg/ml). [b]FC, MIC fold-change in the absence of tunicamycin relative to those in the presence of tunicamycin. Cefotaxime, cefuroxime, and ceftizoxime bind preferentially to PBP2; moenomycin is a GTase inhibitor.

Table S2. Plasmids and strains used in this study.

Strains or plasmids	Relevant genotype or characteristic	Source
Plasmids		
pCL-*lacZ*	*E. coli-S. aureus* shuttle cloning vector, single-copy integration vector in *S. aureus*	(1)
pCL-*lux*	pCL-*lacZ* derivative carrying *luxABCDE*	this study
pCL-*psmα-lacZ*	pCL-*lacZ* derivative carrying *psm*α promoter	this study
pCL-*psmα-lux*	pCL-*lux* derivative carrying *psmα* promoter	this study
pCL-*vraX-lacZ*	pCL-*lacZ* derivative carrying *vraX* promoter	this study
pCL-*vraX-lux*	pCL-*lux* derivative carrying *vraX* promoter	this study
pYJ335	*E. coli-S. aureus* shuttle vector, Cm^r, Erm^r	(2)
pYJ335-1	*A* modified pYJ335 plasmid containing *Asc*I and *Psp*OMI restriction enzyme sites followed by the blunt-end *Eco*RV site of pYJ335	this study
p-*tarO*	pYJ335-1 derivative carrying *tarO* in the downstream of the xyl/tetO promoter	this study
p-*tarO*^{G152A}	p-*tarO* derivative carrying *tarO* which has alanine substitution mutant at the site of glycine residue 152	this study
p-*vraR*	pYJ335-1 derivative carrying *vraR* in the downstream of the xyl/tetO promoter	this study
p-*sgtB*	pYJ335 derivative carrying *sgtB* in the downstream of the xyl/tetO promoter	this study
p-*murJ*	pYJ335 derivative carrying *murJ* in the downstream of the xyl/tetO promoter	this study
p-*mecA*	pYJ335 derivative carrying *mecA* in the downstream of the xyl/tetO promoter	this study
p-*agrA*	pYJ335-1 derivative carrying *agrA* in the downstream of the xyl/tetO promoter	this study
pET28a	T7 *lac* promoter-operator, N-terminal His tag, Kan^r	Novagen
pET28a-His$_6$-*vraR*	pET28a derivative carrying *vraR*	this study
pKOR1	Gene replacement vector for *S. aureus* genes, Amp^r, Cm^r	(3)
pKOR1::*tarO*	pKOR1 derivative, for deletion of *tarO*	this study

pKOR1::*vraR*	pKOR1 derivative, for deletion of *vraR*	this study
pKOR1::*mecA*	pKOR1 derivative, for deletion of *mecA*	this study
pMutin-HA	A suicide vector for introducing mutation in *S. aureus* via a single recombination event	(4)
pMutin-HA-*pbp2'*	pMutin-HA derivative carrying a DNA fragment covering the ribosome binding site region and the first 630 bp (-20 to + 630 of the start codon) of *pbp2*	this study
p-*gfp-pbp2*	pYJ335-1 derivative carrying *gfp-pbp2* fusion in the downstream of the xyl/tetO promoter	this study
S. aureus		
RN4220	Derivative of 8325-4 that accepts plasmids	(5)
USA300 LAC	A CA-MRSA USA300 isolate, sequence type 8 (ST8)	(6)
USA400 MW2	A CA-MRSA USA300 isolate; ST1	(6)
2011-137	A clinic isolate of CA-MRSA lineage ST59	(7)
JE2	*S. aureus* LAC cured of all 3 native plasmids; Erm^s	(8)
JE2:: *vraX-lacZ*	JE2 strain carrying an integration vector pCL-*vraX-lux*	this study
USA300::*psmα-lacZ*	USA300 LAC carrying an integration vector pCL-*psmα-lacZ*	this study
USA300::*psmα-lux*	USA300 LAC carrying an integration vector pCL-*psmα-lux*	this study
USA300::*vraX-lacZ*	USA300 LAC carrying an integration vector pCL-*vraX-lacZ*	this study
USA300::*vraX-lux*	USA300 LAC carrying an integration vector pCL-*vraX-lux*	this study
RN4220::*vraX-lux*	RN4220 carrying an integration vector pCL-*vraX-lux*	this study
Δ*tarO*	*tarO* deletion mutant of USA300 LAC	this study
USA300/pYJ335-1	USA300 LAC carrying plasmid pYJ335-1	this study
Δ*tarO*/pYJ335-1	Δ*tarO* carrying plasmid pYJ335-1	this study
Δ*tarO*/p-*tarO*	Δ*tarO* carrying plasmid p-*tarO*	this study
Δ*tarO*/p-$tarO^{G152A}$	Δ*tarO* carrying plasmid p-$tarO^{G152A}$	this study
Δ*mecA*	*mecA* deletion mutant of USA300 LAC	this study
Δ*mecA*::*vraX-lux*	Δ*mecA* carrying an integration vector pCL-*vraX-lux*	this study
Δ*vraR*	*vraR* deletion mutant of USA300 LAC	this study
Δ*vraR*/pYJ335-1	Δ*vraR* carrying plasmid pYJ335-1	this study

Δ*vraR*::*psmα-lux*	Δ*vraR* carrying an integration vector pCL-*psmα-lux*	this study
Δ*vraR*::*vraX-lux*	Δ*vraR* carrying an integration vector pCL-*vraX-lux*	this study
Δ*vraR*/p-*vraR*	Δ*vraR* carrying plasmid p-*vraR*	this study
Δ*vraR*::*vraX-lacZ*	Δ*vraR* carrying an integration vector pCL-*vraX-lacZ*	this study
USA300-1	USA300 LAC carrying pMutin–HA-*pbp2’*	this study
USA300/p-*gfp-pbp2*	USA300 LAC carrying p-*gfp-pbp2*	this study
Δ*tarO*/p-*gfp-pbp2*	Δ*tarO* carrying p-*gfp-pbp2*	this study
Δ*mecA*/p-*gfp-pbp2*	Δ*mecA* carrying p-*gfp-pbp2*	this dtudy
Δ*agrA*	*agrA* deletion mutant of USA300 LAC	this study
Δ*spa*	*spa* deletion mutant of USA300 LAC	this study
Δ*agrA*Δ*spa*	*agrA* and *spa* double-deletion mutant of USA300 LAC	this study
JE2/pYJ335	JE2 carrying plasmid pYJ335	this study
sgtB	JE2 mutant with mariner-based transposon insertion at 562 bp after the start codon of *sgtB*.	laboratory stock
sgtB::*vraX-lux*	*sgtB* mutant carrying an integration vector pCL-*vraX-lux*	This study
sgtB/pYJ335	*sgtB* carrying plasmid pYJ335	this study
sgtB/p-*sgtB*	*sgtB* carrying plasmid p-*sgtB*	laboratory stock
USA300::*vraX-lux*/pYJ335	USA300::*vraX-lux* carrying plasmid pYJ335	this study
USA300::*vraX-lux*/p-*murJ*	USA300::*vraX-lux* carrying plasmid p-*murJ*	this study
Δ*mecA*::*vraX-lux*/pYJ335	Δ*mecA*::*vraX-lux* carrying plasmid pYJ335	this study
Δ*mecA*::*vraX-lux*/p-*mecA*	Δ*mecA*::*vraX-lux* carrying plasmid p-*mecA*	this study
RN4220::*vraX-lux*/pYJ335	RN4220::*vraX-lux* carrying plasmid pYJ335	this study
RN4220::*vraX-lux*/p-*murJ*	RN4220::*vraX-lux* carrying plasmid p-*murJ*	this study
RN4220::*vraX-lux*/p-*mecA*	RN4220::*vraX-lux* carrying plasmid p-*mecA*	this study
DH5α	for DNA cloning	laboratory stock
BL21 star (DE3)	F^-*ompT hsdS*$_B$ (r_B^- m_B^-) *gal dcm met* (DE3)	laboratory stock

Amp^r, ampicillin resistance; Cm^r, chloramphenicol resistance; Erm^r, erythromycin resistance; Erm^s, erythromycin sensitive; Kan^r, kanamycin resistance

Table S3. Primers used in this study.

Primers	Sequence (5′to 3′)	Purpose
psmα-pro-F	CCGGAATTCCACTGCATAACCTCCTTATTTCTAA	for pCL-*psmα-lacZ*
psmα-pro-R	CGGGGTACCTAAGATTACCTCCTTTGCTTATGAGT	
vraX-pro-F	CCGGAATTCTGGATCACGGTGCATACAAC	for pCL-*vraX-lacZ*
vraX-pro-R	CGGGGTACCCCTATATTACCTCCTTTGCTACTCTAT	
luxABCDE-F	CCGGAATTCGAGCTCGAGCGCCACGTGATGAAGCAAGAGGAGGACTCTC	for pCL-*lux*
luxABCDE-R	CGGGGTACCGTCGACTTAACTATCAAACGCT	
psmα-pro-*lux*-R	CCGCTCGAGCTTTGCTTATGAGTTAACTTCATTG	for pCL-*psmα-lux*
vraX-pro-*lux*-R	CCGCTCGAGCCTTTGCTACTCTATGGTTATATTATAA	for pCL-*vraX-lux*
tarO-up-F	GGGGACAAGTTTGTACAAAAAAGCAGGCTGTGAATGACAACTGAGAACTCTTC	for pKOR1::*tarO*
tarO-up-R	CCATACAGCTATGCTTTCATTCCTTATTCACCTTCATCGATATTAATTG	
tarO-down-F	GGAATGAAAGCATAGCTGTATGG	
tarO-down-R	GGGGACCACTTTGTACAAGAAAGCTGGGTGTCACACTTAATGGCGCTATTTG	
tarO-F	ACAATTAATATCGATGAAGGTGAATAA	for p-*tarO*
tarO-R	AGGGGGCCCACAGCTATGCTTTCATTCCCTATT	
vraR-up-F	GGGGACAAGTTTGTACAAAAAAGCAGGCTATTGGTTCAATGCTCATCTTAGTA	for pKOR1::*vraR*
vraR-up-R	TCTTAATTCGATATGAACTATTGAATTAACCACAAACAATACTTTAATCGTCA	
vraR-down-F	TTAATTCAATAGTTCATATCGAATTAAGA	
vraR-down-R	GGGGACCACTTTGTACAAGAAAGCTGGGTATGTTGCTTTTCCTATTAACATTATTA	
vraR-F	CCTTTAAATAAGGAGGATTCGTATG	for p-*vraR*
vraR-R	AGGGGGCCCTTCGATATGAACTATTGAATTAAATTATG	
His-*vraR*-F	CGCGGATCCATGACGATTAAAGTATTGTTTGTGG	for pET28a-His$_6$-*vraR*
His-*vraR*-R	CCGCTCGAGTTCGATATGAACTATTGAATTAAATTATG	
agrA-up-F	GGGGACAAGTTTGTACAAAAAAGCAGGCTGTATTTTGTCAAAATCAAATGGTATT	for pKOR1::*agrA*
agrA-up-R	GCCGTTAACTGACTTTATTATCTTAACATTCACATCCTTATGGCTAGTT	
agrA-down-F	TAAGATAATAAAGTCAGTTAACGGC	
agrA-down-R	GGGGACCACTTTGTACAAGAAAGCTGGGTAAACATTTACGAAGCAAATTGGT	

spa-up-F	GGGGACAAGTTTGTACAAAAAAGCAGGCTAAGC GTCGACTTTCTTAATTACA	for pKOR1::*spa*
spa-up-R	TCTATCGTTGTGTATTGTTTGTTTCTGTATGTATTT GTAAAGTCATCATAAT	
spa-down-F	AAACAAACAATACACAACGATAGA	
spa-down-R	GGGGACCACTTTGTACAAGAAAGCTGGGTATTG ATACGTTTAACTTAAGTGGAG	
mecA-up-F	GGGGACAAGTTTGTACAAAAAAGCAGGCTAAGA ACTTTATGTCCCGGACTCAT	for pKOR1::*mecA*
mecA-up-R	CTTTACCTGAGATTTTGGCATTGTCGACAACTACA ACTATTAAAATAAGTGG	
mecA-down-F	ACAATGCCAAAATCTCAGGTAAAG	
mecA-down-R	GGGGACCACTTTGTACAAGAAAGCTGGGTGTGC GATTAATGTGATAATACAGTTACG	
pYJ335-PCR-F	GATATCCCCTTGGCGCGCCAAAGGGGGCCCCCTG AATTCGGAGGCATATC	for pYJ335-1
pYJ335-PCR-R	GGGCCCCCTTTGGCGCGCCAAGGGGATATCAAGC TTATTTTAATTATACTCTATCA	
EMSA-*spa*-F	TATTACGCAAGTGTGCTGTATTCTA	for EMSA of *spa*
EMSA-*spa*-R	ATTAATACCCCCTGTATGTATTTGTAA	
EMSA-*vraX*-F	GAAAATTTCATACATATCGAACAATAC	for EMSA of *vraX*
EMSA-*vraX*-R	AACCTATATTACCTCCTTTGCTACTC	
EMSA-*vraRS*-F	GGTCCGATTTTAACGACAAAG	for EMSA of *vraRS*
EMSA-*vraRS*-R	TGAAATGACGCATTGATTGTG	
EMSA-*coa*-F	GTGTTGTCATGCTTTGTTACTCC	for EMSA of *coa*
EMSA-*coa*-R	GCGCCTAGCGAAATTATTTGC	
FAM-*spa*-R	FAM-ATTAATACCCCCTGTATGTATTTGTAA	for footprinting assay of *spa*
FAM-*vraX*-R	FAM-AACCTATATTACCTCCTTTGCTACTC	for footprinting assay of *vraX*
G152A-F	GCAATTAACTTAATTGATGCTCTCGATGGTTTGG	for p-*tarO*^{G152A}
G152A-R	CCAAACCATCGAGAGCATCAATTAAGTTAATTGC	
spa-RT-F	GAAAAAGAAAAACATTTATTCAATTCG	for qRT-PCR of *spa*
spa-RT-R	AGGCATATTTAAGACTTGATAAAAAGC	
psmα3-RT-F	GCCATTCACATGGAATTCGT	for qRT-PCR of *psmα3*
psmα3-RT-R	GATTAGTTGTTACCTAAAAATTTACCAAG	
psmβ1-RT-F	ATGGAAGGTTTATTTAACGCAAT	for qRT-PCR of *psmβ1*
psmβ1-RT-R	AATCCGAATAATTTACCTAATAAACC	
RNAIII-RT-F	GGAAGGAGTGATTTCAATGG	for qRT-PCR of RNAIII
RNAIII-RT-R	TTCACTGTGTCGATAATCCA	
vraX-RT-F	ATGATTATTTATCGACAGTATCACCAT	for qRT-PCR of *vraX*
vraX-RT-R	AGAGCAATTTGAATATTTCAGTATCAC	
pbp2-RT-F	GATTTAAACTTAGCGGAAGAAGC	for qRT-PCR of *pbp2*

pbp2-RT-R	TGTTTTTACGATCTTCAGCAGC	
saeR-RT-F	CACTTACTGATCGTGGATGATGA	for qRT-PCR of *saeR*
saeR-RT-R	ATCATTTGATAGTAAAGAAATTGCTTC	
saeS-RT-F	TAGAAGTCAAATCATTATTGGCGT	for qRT-PCR of *saeS*
saeS-RT-R	CAGCTTGTAATTATTGTCGTTAAGG	
pmtA-RT-F	GAATGCCATAGAATTAAGTAATGTT	for qRT-PCR of *pmtA*
pmtA-RT-R	CTTATTATTGTGGTTTTACCAGCG	
16S-RT-F	GGCAAGCGTTATCCGGAATT	for qRT-PCR of 16S rRNA
16S-RT-R	GTTTCCAATGACCCTCCACG	
P*spac-pbp2*-F	CCCAAGCTTGATGAAAGTGAGGACCGCGT	for pMutin-HA-*pbp2'*
P*spac-pbp2*-R	GCGGGTACCATACTTAGCAGCAGCTTTAATACCTG	
gfp-F	GATGAAAGTGAGGACCGCGTATGTCAAAAGGAG AAGAATTATTTAC	for p-*gfp-pbp2*
gfp-R	TGAGAAGATCCTTTGTTTTCCGTCTTATATAATTCAT CCATTCCGTG	
pbp2-F	ACGGAAAACAAAGGATCTTCTCA	for pMutin-HA-*pbp2'*
pbp2-R	AGGGGGCCCAGTGGATTAGTTGAATATACCTGTTA ATC	
sgtB-F	TTAAAAGAAGGAGCAAACGCAT	for p-*sgtB*
sgtB-R	ATGCAAGTATTTAACGATTTAATTGTG	
mecA-F	CTACAAATGTAGTCTTATATAAGGAGTATATTG	for p-*mecA*
mecA-R	TTACGGATTGCTTCACTGTTTT	
murJ-F	AATTAAAATAAGCTTATGAGATAGGGAGATTCGTA	for p-*murJ*
murJ-R	TATGCCTCCGAATTCTCATCGTAAAAACCTAACTC	
agrA-F	CAACTAGCCATAAGGATGTGAATG	for p-*agrA*
agrA-R	AGGGGGCCCGCCGTTAACTGACTTTATTATCTTATT A	

SI References

1. Sun F, *et al.* (2010) In the Staphylococcus aureus two-component system sae, the response regulator SaeR binds to a direct repeat sequence and DNA binding requires phosphorylation by the sensor kinase SaeS. *J Bacteriol* 192(8):2111-2127.
2. Ji Y, Marra A, Rosenberg M, & Woodnutt G (1999) Regulated antisense RNA eliminates alpha-toxin virulence in Staphylococcus aureus infection. *J Bacteriol* 181(21):6585-6590.
3. Bae T & Schneewind O (2006) Allelic replacement in Staphylococcus aureus with inducible counter-selection. *Plasmid* 55(1):58-63.
4. Grundling A & Schneewind O (2007) Synthesis of glycerol phosphate lipoteichoic acid in Staphylococcus aureus. *Proceedings of the National Academy of Sciences of the United States of America* 104(20):8478-8483.
5. Kreiswirth BN, *et al.* (1983) The toxic shock syndrome exotoxin structural gene is not detectably transmitted by a prophage. *Nature* 305(5936):709-712.
6. Bubeck Wardenburg J & Schneewind O (2008) Vaccine protection against Staphylococcus aureus pneumonia. *The Journal of experimental medicine* 205(2):287-294.

7. Li M, *et al.* (2016) Increased Community-Associated Infections Caused by Panton-Valentine Leukocidin-Negative MRSA, Shanghai, 2005-2014. *Emerg Infect Dis* 22(11):1988-1991.
8. Fey PD, *et al.* (2013) A genetic resource for rapid and comprehensive phenotype screening of nonessential Staphylococcus aureus genes. *mBio* 4(1):e00537-00512.
