# Supplementary material for: Modulation of MRSA virulence gene expression by the wall teichoic acid enzyme TarO
Source: Nat Commun. 2023 Mar 22;14:1594. doi: 10.1038/s41467-023-37310-5 (PMC10032271; doi:10.1038/s41467-023-37310-5)
Supplement: Supplementary file 3 — Description of Additional Supplementary Files [file 41467_2023_37310_MOESM3_ESM.pdf]

Description of Additional Supplementary Files

File Name: Supplementary Data 1
Description: Screening compounds and potential psmα-lacZ inhibitors.

File Name: Supplementary Data 2
Description: RNA-seq analysis.
