# Supplementary material for: Modulation of MRSA virulence gene expression by the wall teichoic acid enzyme TarO
Source: Nat Commun. 2023 Mar 22;14:1594. doi: 10.1038/s41467-023-37310-5 (PMC10032271; doi:10.1038/s41467-023-37310-5)
Supplement: Supplementary file 6 — Reporting Summary [file 41467_2023_37310_MOESM6_ESM.pdf]

nature portfolio

Corresponding author(s): Lefu Lan

Last updated by author(s): Feb 25, 2023

Reporting Summary

Nature Portfolio wishes to improve the reproducibility of the work that we publish. This form provides structure for consistency and transparency in reporting. For further information on Nature Portfolio policies, see our Editorial Policies and the Editorial Policy Checklist.

Statistics

For all statistical analyses, confirm that the following items are present in the figure legend, table legend, main text, or Methods section.

n/a	Confirmed	
☐	☒	The exact sample size (*n*) for each experimental group/condition, given as a discrete number and unit of measurement
☐	☒	A statement on whether measurements were taken from distinct samples or whether the same sample was measured repeatedly
☐	☒	The statistical test(s) used AND whether they are one- or two-sided<br>*Only common tests should be described solely by name; describe more complex techniques in the Methods section.*
☐	☒	A description of all covariates tested
☒	☐	A description of any assumptions or corrections, such as tests of normality and adjustment for multiple comparisons
☐	☒	A full description of the statistical parameters including central tendency (e.g. means) or other basic estimates (e.g. regression coefficient) AND variation (e.g. standard deviation) or associated estimates of uncertainty (e.g. confidence intervals)
☐	☒	For null hypothesis testing, the test statistic (e.g. *F*, *t*, *r*) with confidence intervals, effect sizes, degrees of freedom and *P* value noted<br>*Give P values as exact values whenever suitable.*
☒	☐	For Bayesian analysis, information on the choice of priors and Markov chain Monte Carlo settings
☒	☐	For hierarchical and complex designs, identification of the appropriate level for tests and full reporting of outcomes
☒	☐	Estimates of effect sizes (e.g. Cohen's *d*, Pearson's *r*), indicating how they were calculated

Our web collection on statistics for biologists contains articles on many of the points above.

Software and code

Policy information about availability of computer code

Data collection	Microscopy images were obtained by LEICA TCS SP8 confocal microscopy (100 X objective, zoom 4.5) and LAS X software.<br>RNA-seq was performed by HiSeq X Ten system (Illumina).<br>Chemiluminescent light was detected by Tanon-5200 multi (Tanon Science & Technology Co., Ltd.).<br>Promoter activities were measured using a Synergy 2 Multi-Mode Microplate Reader (Biotek).
Data analysis	Statistical analysis was with Graph Pad Prism 7.0<br>Densitometric analysis of gel lanes was executed with the ImageJ software (version 1.4.3.67).<br>Deconvolution treatment of the images was performed using Huygens software (Huygens Professional).<br>For RNA-seq analysis, bacterial RNA-seq reads were mapped to the S. aureus USA300 LAC genome (GenBank: CP055225.1) using Bowtie 2 (version 2.3.2) and DESeq2 Package was used for the determination of differentially expressed genes (DEGs).<br>Electropherograms of DNase I footprinting assay were analyzed with GeneMarker version 1.91 (Applied Biosystems).

For manuscripts utilizing custom algorithms or software that are central to the research but not yet described in published literature, software must be made available to editors and reviewers. We strongly encourage code deposition in a community repository (e.g. GitHub). See the Nature Portfolio guidelines for submitting code & software for further information.

Data

Policy information about availability of data

All manuscripts must include a data availability statement. This statement should provide the following information, where applicable:

- Accession codes, unique identifiers, or web links for publicly available datasets
- A description of any restrictions on data availability
- For clinical datasets or third party data, please ensure that the statement adheres to our policy

S. aureus USA300 LAC genome can be found at National Center for Biotechnology Information with the accession number of CP055225.1 (https://www.ncbi.nlm.nih.gov/nuccore/CP055225.1). RNA-Seq data have been deposited in the NCBI Sequence Read Archive (SRA), with accession number PRJNA746457 (https://www.ncbi.nlm.nih.gov/bioproject/?term=PRJNA746457). Other data are included in the article and/or the supplementary information and source data file. Source data are provided with this paper.

Human research participants

Policy information about studies involving human research participants and Sex and Gender in Research.

Reporting on sex and gender	n/a
Population characteristics	n/a
Recruitment	n/a
Ethics oversight	n/a

Note that full information on the approval of the study protocol must also be provided in the manuscript.

Field-specific reporting

Please select the one below that is the best fit for your research. If you are not sure, read the appropriate sections before making your selection.

☒ Life sciences ☐ Behavioural & social sciences ☐ Ecological, evolutionary & environmental sciences

For a reference copy of the document with all sections, see nature.com/documents/nr-reporting-summary-flat.pdf

Life sciences study design

All studies must disclose on these points even when the disclosure is negative.

Sample size	No statistical methods were used to determine sample sizes. The sample size was determined according to our experience and literature reporting similar experiments (e.g., Nat Commun. 2022 Nov 14;13(1):6909; Nat Chem Biol. 2016 Mar;12(3):174-9; Proc Natl Acad Sci U S A. 2010 Nov 2;107(44):18991-6).
Data exclusions	No data were excluded from the analysis.
Replication	Each experiment was performed at least three biological replicates and all attempts at replication are successful.
Randomization	For infection experiments, G. mellonella larvae or mice were infected with S. aureus randomly and allocated into different groups. Other samples, e.g., the bacteria culture samples and biochemical samples were maintained under the same environment and also allocated randomly into different groups.
Blinding	Galleria mellonella larvae infection experiments and fluorescence microscopy assays were performed by an experimenter who was blinder to samples. Enumeration of the bacterial CFUs in the murine infection samples were also performed blindly. The investigators did not know the sample information. Other experiments were not blindly performed since the results could be directly obtained from visual observations or by instrument measurements.

Reporting for specific materials, systems and methods

We require information from authors about some types of materials, experimental systems and methods used in many studies. Here, indicate whether each material, system or method listed is relevant to your study. If you are not sure if a list item applies to your research, read the appropriate section before selecting a response.

Materials & experimental systems

n/a	Involved in the study
☐	☒ Antibodies
☒	☐ Eukaryotic cell lines
☒	☐ Palaeontology and archaeology
☐	☒ Animals and other organisms
☒	☐ Clinical data
☒	☐ Dual use research of concern

Methods

n/a	Involved in the study
☒	☐ ChIP-seq
☒	☐ Flow cytometry
☒	☐ MRI-based neuroimaging

Antibodies

Antibodies used

For immunoblotting following antibodies were used: anti-SpA (Abcam, Catalog no. ab60206, 1:5000 dilution), anti-SrtA (Abcam, Catalog no. ab13959, 1:2500 dilution), anti-rabbit IgG conjugated to horseradish peroxidase (HRP) (CoWin Biosciences, Catalog no. CW0103, 1:5000 dilution).

Validation

All the above antibodies are commercially available and routinely used by our lab and many others.
anti-SpA: https://www.abcam.cn/protein-a-antibody-ab60206.html
anti-SrtA: https://www.abcam.cn/sortase-a-antibody-ab13959.html
anti-Rabbit IgG: https://cwbio.com/goods/index/id/10119

Animals and other research organisms

Policy information about studies involving animals; ARRIVE guidelines recommended for reporting animal research, and Sex and Gender in Research

Laboratory animals

6-8 weeks old female BALB/c mice (about 18-20 g) were purchased from Shanghai Jiesijie Laboratory Animal Co., Ltd (https://shminxing083596.11467.com/). All mice were housed in the specific pathogen-free facility and were given an ad libitum diet. Dark and light were cycled every 12 hrs. The ambient temperature was 20–26°C, and the humidity was 40–60%.

G. mellonella larvae (250-300 mg each) in the last larval stage, which is about 6 weeks old, were were purchased (https://yumichong321.etlong.com/) and used in this study.

Wild animals

This study did not involve wild animals.

Reporting on sex

Female mice were used in this study because it is easier to keep the female mouse together. Male mouse however, tend to fight against each other if kept together. Sex was not considered in the study design.

Field-collected samples

No field-collected sample was used in this study.

Ethics oversight

Mouse infection experiments were performed in strict accordance with the regulations for the Administration of Affairs Concerning Experimental Animals approved by the State Council of People's Republic of China (11-14-1988). The protocols of animal study were reviewed and approved by the Institutional Animal Care and Use Committee (IACUC) of the Shanghai Public Health Clinical Center (permit 2013P201) and were performed in accordance with the relevant guidelines and regulations. The laboratory animal usage license number is SYXK-HU-2010-0098, certified by Shanghai Committee of Science and Technology.

Note that full information on the approval of the study protocol must also be provided in the manuscript.
